# The Valorization of Wastes and Byproducts from Cruciferous Vegetables: A Review on the Potential Utilization of Cabbage, Cauliflower, and Broccoli Byproducts

**DOI:** 10.3390/foods13081163

**Published:** 2024-04-11

**Authors:** Tharushi S. Shinali, Yiying Zhang, Moater Altaf, Assa Nsabiyeze, Zixin Han, Shuyuan Shi, Nan Shang

**Affiliations:** 1 College of Engineering, China Agricultural University, Beijing 100083, China; tharushisanitha@cau.edu.cn (T.S.S.); z_yeeong_zyy@outlook.com (Y.Z.); assansabiyeze1@gmail.com (A.N.); h919hzx@163.com (Z.H.); 2 College of Biological Sciences, China Agricultural University, Beijing 100083, China; moateraltaf6@gmail.com; 3 Key Laboratory of Functional Dairy, College of Food Science and Nutritional Engineering, China Agricultural University, Beijing 100083, China; ssy08300929@163.com; 4 Key Laboratory of Precision Nutrition and Food Quality, Department of Nutrition and Health, China Agricultural University, Beijing 100083, China

**Keywords:** cruciferous, valorization, vegetable waste, value-added products, food industry applications

## Abstract

The management of vegetable waste and byproducts is a global challenge in the agricultural industry. As a commonly consumed vegetable crop, cruciferous vegetables marked higher amounts of wastage during their supply chain processes, with a significant contribution from cabbage, cauliflower, and broccoli. Therefore, the sustainable and resource-efficient utilization of discarded materials is crucial. This review explores potential applications of cruciferous vegetable waste and byproducts, spotlighting cabbage, cauliflower, and broccoli in food, medicinal, and other industries. Their significance of being utilized in value-added applications is addressed, emphasizing important biomolecules, technologies involved in the valorization process, and future aspects of practical applications. Cabbage, cauliflower, and broccoli generate waste and low-processing byproducts, including leaves, stems, stalks, and rot. Most of them contain high-value biomolecules, including bioactive proteins and phytochemicals, glucosinolates, flavonoids, anthocyanins, carotenoids, and tocopherols. Interestingly, isothiocyanates, derived from glucosinolates, exhibit strong anti-inflammatory and anticancer activity through various interactions with cellular molecules and the modulation of key signaling pathways in cells. Therefore, these cruciferous-based residues can be valorized efficiently through various innovative extraction and biotransformation techniques, as well as employing different biorefinery approaches. This not only minimizes environmental impact but also contributes to the development of high-value-added products for food, medicinal, and other related industries.

## 1. Introduction

Agricultural production and agro-industrial processing generate substantial waste, contributing to adverse environmental and economic concerns [1]. The emission of greenhouse gases and the economic cost of waste management are major challenges. Despite these challenges, agricultural byproducts, rich in bioactive compounds, proteins, essential oils, starch, and more, offer the potential for functional food uses, biomaterial recovery, and biofuel development [2,3]. According to world agricultural produce data, around 33% of the consumable portions of food that are meant for human consumption are lost or wasted [4]. Therefore, recently, more research has prioritized the development of appropriate treatments and technologies to enhance the retrieval of valuable materials from waste and byproducts and optimize their processing and storage conditions, ensuring their potential use in different applications.

Cruciferous crops, commonly known as *Brassicas* or crucifers, are grown worldwide as temperate crops, including cabbage, cauliflower, broccoli, brussels sprouts, kale, and radish. These vegetables are rich in nutritious and other health-beneficial components, including dietary fiber, protein, vitamins, minerals, and significant bioactive compounds. In particular, glucosinolates, anthocyanins, flavonoids, coumarins, and bioactive phenolic acids are major beneficial biomolecules present in these vegetables, which provide anticancer, antioxidant, anti-inflammatory, and antimicrobial effects [5,6,7,8]. However, with the increase in the global production of cruciferous crops over the past years, a considerable amount of cruciferous waste and byproducts during the supply chain is also generated. According to the Food and Agriculture Organization (FAO), cabbage and cauliflower, known as the major cruciferous vegetables, accounted for an annual global production of 71.8 and 25.3 million tons in 2018, respectively. In 2019, it was reported that the global production of cruciferous crops was 97 million tons, along with a significant amount of waste materials. Among the global producers, China is marked as the major producer, with 33.9 million tons of cabbage and 10.3 million tons of cauliflower worldwide. In 2022, the FAO reported that the global production of cruciferous crops, including cabbage, reached a total of 72,604 kilotons. This represents an increase of 0.876% compared to the previous year and a 6.53% increase compared to the data from 10 years ago. Consequently, during different stages of the vegetable supply chain, cruciferous vegetables generate waste and low-value processing byproducts throughout the world. 

Therefore, this review centers on the valorization of cruciferous byproducts, with a special focus on cauliflower, cabbage, and broccoli. The aim is to convert these byproducts into value-added components for diverse applications, including functional ingredients in food, nutraceutical products, medicinal applications, and other potential uses. Additionally, we delve into the identification of significant biomolecules, their potential, and the essential technological aspects for utilizing these compounds in various applications. 

## 2. Compositional Significance of Cruciferous Vegetable Waste and Byproducts 

### 2.1. Varieties of Cruciferous Vegetable Waste and Byproducts

During different stages of the vegetable supply chain from farmlands to consumers, cruciferous vegetables, including cabbage, cauliflower, and broccoli, generate waste and low-value processing byproducts in the form of rot, stems, stalks, and leaves throughout the world. During the preparation process, outer leaves, stems, roots, and peels are often discarded as waste material. Also, industrial processing can generate different cruciferous byproducts such as stem and lead residuals and cabbage cores. The total waste generated from cabbages and cauliflowers averages between 30 and 50% [9], with Korea, the world’s fourth-largest cabbage producer, generating up to 30% of the waste from total cabbage production [10]. Approximately 60–75% of the world’s broccoli production is discarded as waste during harvesting [11]. 

Generally, only a small portion of the broccoli plant, florets together with attached stalks and sprouts, have a market value that is less than 30% of the entire broccoli plant [12]. Its leaves, stalks, and inflorescences can be identified as commercially underutilized and mostly discarded. In the industry, low-quality, non-marketable broccoli seed cultivars are also discarded as industrial byproducts, which can be employed to extract bioactive components [13]. The rapid growth of cauliflower cultivation during the past few years also contribute to generating a higher amount of cauliflower byproducts [14]. Accordingly, the large green leaves that surround the cauliflower head and thick stalks and stems of cauliflower are often discarded. Cabbage leaves, stems, and outer leaves are also discarded as byproducts during the cabbage supply chain process [15]. The generation of a considerable amount of discarded waste and byproducts from these cruciferous vegetables has a huge impact on the economic as well as the environmental aspects. However, these byproducts still contain beneficial components that can be valorized through value addition. 

### 2.2. Nutritional and Phytochemical Composition

Most of the fruit- and vegetable-derived waste materials and processing byproducts, mainly discarded or used as animal feed, contain abundant high-value compounds. Therefore, many studies attracted their focus on the recovery of those materials that have the potential to be used in value-added applications. Consequently, edible cruciferous vegetables are rich in nutritious and other health-beneficial components, including dietary fiber, protein, vitamins, minerals, and significant bioactive compounds. Therefore, currently, the research focuses on valorizing cruciferous vegetable waste and byproducts, taking advantage of the possession of high-value components. However, the nutritional and phytochemical profiles vary between various cultivars due to the differences in the genetic composition of the crop, climatic conditions, and agronomic conditions [16]. 

#### 2.2.1. Nutritional Profile

Cruciferous vegetables have higher nutritional value, containing diverse nutrients, including minerals, dietary fiber, vitamins, and other nutrients. The proximate nutritional compositions of different cruciferous vegetables are given in Table 1. The content varies among different botanical parts of the crop plant. Li and colleagues [17] report the nutritional value of different botanical parts of broccoli, including edible and underutilized parts. Accordingly, broccoli stalks and leaves are an excellent source of minerals such as calcium, potassium, zinc, and iron. Although there are variations in mineral content in various parts of the crop, broccoli leaves are a rich source of minerals. Important plant proteins, including albumin, prolamin, glutelin, and globulin, as well as amino acids, including tyrosine, glutamic acid, glutamine, and asparagine, are also abundantly found in broccoli byproducts. Dietary fiber is also an important component in various broccoli parts [17], and it is also reported that the dietary fiber content and the cell wall-based dietary fiber composition in broccoli stems were found to be changed during storage [18]. Cauliflower byproducts, including stems and leaves, are reported to contain beneficial functional components such as dietary fiber, leaf proteins, and vitamin C [19]. Some of these protein hydrolysates show important antioxidant and regulatory effects on glucose metabolism. 

#### 2.2.2. Phytochemical Composition

Cruciferous vegetables are particularly high in phytochemical substances known as bioactive secondary metabolites, which exist naturally in these plants. Accordingly, many heterogeneous phytochemicals, such as glucosinolates and phenolic constituents, including flavonoids, anthocyanins, carotenoids, and tocopherols, are abundant in cruciferous vegetables. Table 2 depicts various significant biomolecules in specific cruciferous vegetable byproducts characterized by different methods. Kaempferol, quercetin, and isorhamnetin are commonly found flavonoids in cruciferous crop plants [22]. They can be found as either O-glycosides, which are conjugated to glucose, or chemical compounds, which are acylated by hydroxycinnamic acids. These chemicals have the ability to interact with a variety of molecular targets within cells. The interaction of numerous elements, such as germination, environmental conditions, and nutrient delivery in the crops, determine the synthesis, diversity, and amount of phytochemicals in these plants and the chemical composition of each component responsible for varied bioactive properties [23]. 

**Figure 1:**
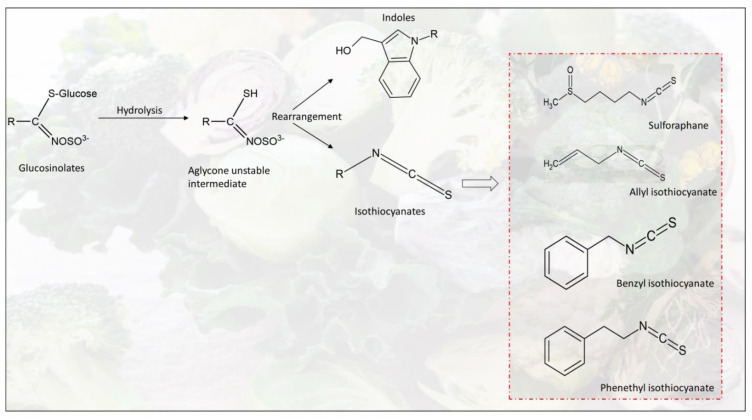
Formation of major bioactive indoles and isothiocyanates through glucosinolates hydrolysis.

Crop plants from the family *Cruciferae*, such as cauliflower, broccoli, cabbage, rapeseed–mustard, and Brussels sprouts, are well known to contain glucosinolates. These glucosinolates are sulphur-containing secondary metabolites where different types of aliphatic, aromatic, or indolyl glucosinolates are present with the basic structure, which is composed of a β-D glucose moiety linked to a sulphated thiohydroximate. Glucosinolates act as precursors of bioactive compounds such as isothiocyanates and indoles in cruciferous vegetables [24]. Figure 1 shows the formation of major bioactive compounds, including indoles and a few significant isothiocyanates, through the hydrolysis of glucosinolates. In addition to glucosinolates, cauliflower byproducts are also rich in vitamin C, carotenoids, and phenolic compounds such as kaempferol, quercetin, and hydroxycinnamic acids [19]. According to a study, cauliflower byproducts (leaves and stems) contain phenolic compounds with potential antioxidant activity, and it is also reported that byproducts from cauliflower contain a three-fold higher amount of flavanols than in other cruciferous species [25]. A total of 28 polyphenol compounds from cauliflower byproducts, which are naturally present in the plant and produced after acid or alkali hydrolysis, were characterized using a high-performance liquid chromatograph coupled with online mass spectrometry with an electrospray ionization source (HPLC-DAD-MS/MS ESI) [25]. Another study revealed that cut-offs from cauliflower, white cabbage, and broccoli stems show excellent antioxidant activity, while broccoli byproducts indicate the highest antioxidant potency among them [26]. Red cabbage, including the wastes generated during processing throughout the food supply chain, is identified as a rich source of anthocyanin, which is a natural pigment with higher antioxidant properties and health-beneficial effects [27]. The content of naturally occurring chlorophyll green pigments (5258.8 μg/g DW) and carotenoids (1095.0 μg/g DW), which are mainly responsible for red, orange, and yellow colors, are significantly higher in broccoli leaves than in florets and stalks [17]. 

**Table 2:** Significant compounds found in cruciferous waste/byproducts.

Source	Characterization	Significant Compounds	Reference
Cauliflower stem and leaves	Ultrasound-assisted extraction (UAE)	Isothiocyanates	[28]
Cauliflower byproducts	High-resolution Q-TOF-LC–MS/MS	ACE inhibitory peptide	[29]
Green and red cabbage	High-performance liquid chromatography–mass spectrometry (HPLC-MS) and HPLC	6 aliphatic glucosinolates (glucoiberin, glucoiberverin, sinigrin, gluconapin, glucoraphanin, and pro-goitrin), three indolyl glucosinolates (glucobrassicin, neoglucobrassicin, and 4-methoxy glucobrassicin), and one aromatic glucosinolates (gluconasturtiin)	[24]
Cauliflower byproducts (outer leaves)	Ultra(high)-pressure liquid chromatography–electrospray ionization-time-of-flight-ion mobility-high definition mass spectrometry	19 flavonoid glycosides (8 non-acylated and 11 acylated), major aglycones: kaempferol and quercetin, major phenolic acids: sinapic acid and ferulic acid	[30]
Industrial broccoli discards	Ultra-high performance liquid chromatography–MS/MS (UPLC MS/MS)	Aliphatic and indolic glucosinolates, flavonoids (kaempherol-3-o-sophoroside, quercetin-3-diglucoside-7-glucoside), hydroxycinnamic acids	[13]
Fermented broccoli stalk byproduct	Liquid Chromatography–Electrospray Ionization-Triple Quadrupole Mass Spectrometry (LC-ESI-QqQ-MS/MS)	Glucoerucin, indolic glucosinolates (glucobrassicin, 4-methoxy-glucobrassicin, and 4-hidroxy-glucobrassicin), phenolic acids, and flavonoids (sinapic acid, 4-O-feruloyl quinic acid, and quercetin-3-O-diglucoside)	[31]

### 2.3. Significant Characteristics of Cruciferous Byproducts

Packed with the above-discussed essential nutritional components and significant bioactive compounds, these cruciferous vegetables and their byproducts offer a wide range of beneficial effects in a number of applications. Therefore, cruciferous vegetable, including broccoli, derivatives contribute to improving digestion, enhancing metabolic health, and reducing the risk of chronic diseases. Furthermore, different bioactive compounds in these waste and byproducts were shown to have anti-inflammatory, antimicrobial, antioxidant, and anticancer properties [32,33,34,35]. 

According to the literature, protein hydrolysates derived from cauliflower byproducts reveal their antioxidant properties, inhibitory effects on Angiotensin I-converting enzyme (ACE) in cell-free systems as well as their role in regulating glucose consumption and glycogen content in HepG2 cells [19]. Broccoli byproducts have the potential to act as co-delivery vehicles for polyunsaturated oil together with epigallocatechin gallate to enhance their antioxidant properties as well as increase stability during gastrointestinal digestion [32]. A recent research work studied better thickening and emulsifying abilities of pectin extracted from broccoli stalks to be used as an alternative pectin source in the food industry [11]. Also, red cabbage is found to have significant phytochemicals with beneficial therapeutic effects against hypercholesterolemia and hypertriglyceridemia [33]. Another study evaluated broccoli byproducts, including leaf, inflorescence, and stem, for their bioactive compounds with great antioxidant, ACE inhibitory, and antimicrobial activities [34]. Due to the higher contents of fatty acid derivatives, they showed better inhibitory effects against *Bacillus cereus*, *Staphylococcus aureus*, and *Listeria innocua*, the inhibitory percentage ranging from 56% to 94%. However, there can be significant differences in the exhibited bioactivities based on different climatic and agronomic conditions. 

With various beneficial effects, cruciferous vegetables and their byproducts are poised to be employed as functional ingredients in food products and nutraceuticals, providing health benefits and functional advantages across various industries.

## 3. Important Technological Aspects in Byproduct Valorization

### 3.1. Influence of Processing Conditions to Be Used in Potential Applications

Postharvest ultraviolet (UV) treatment, a cost-effective technology, can boost the production of health-promoting compounds in cruciferous agricultural waste and byproducts. Recent studies explored the UV-B and UV-C radiation on broccoli leaves and stalks byproducts during storage (72 h at 15 °C), revealing elevated levels of phenolic content, antioxidant capacity, and glucoraphanin/glucobrassicin [35]. Moreover, postharvest irradiation with UV-B and sunlight was found to be effective in increasing the kaempferol and quercetin glycosides of cabbage leafy waste [36]. In contrast to the damage caused by higher doses of UV-B radiation (280–320 nm) in plant materials, the optimum lower doses of UV-B radiation are reported to enhance the biosynthesis of plant secondary metabolites, including beneficial bioactive compounds mainly due to the morphological, physiological, and the molecular conformational changes caused by the UV-B light exposure [37]. 

Different processing conditions applied during the processing of vegetable byproducts into value-added products may have various effects on their bioactivity. In producing antioxidant-rich dietary fiber powder from cabbage outer leaves, steam blanching was identified to better retain antioxidant bioactive compounds of cabbage outer leaves compared to water blanching [38]. Steam blanching is effective since the blanching time is shorter than in water blanching, and the loss of water-soluble bioactive compounds into the blanched medium is also low. However, depending on various processing parameters, the drying process results in the loss of phytochemicals during the fruit and vegetable waste valorization process [38,39]. In contrast, it is found that drying under high temperatures and short time conditions may result in enhanced antioxidant activity of broccoli with the release of potent antioxidant components from the cell matrix and highly active polyphenols with an intermediate state of oxidation released during polyphenol oxidation [40].

### 3.2. Technologies Involved in Waste Utilization 

The valorization of cruciferous vegetable waste is crucial in maximizing resource utilization as well as minimizing environmental impact. Different advanced and conventional technological methods are mainly employed in valorization with respect to their potential for sustainable resource recovery and value creation. Table 3 provides more details about the use of different technologies for the valorization of cabbage, cauliflower, and broccoli for potential applications and the production of bioproducts with regard to specific cruciferous waste/byproduct types.

Among various advanced technologies, biorefinery approach, anaerobic digestion, and pyrolysis can be considered as commonly employed methods in cruciferous vegetable waste valorization. The development of biobased products while reducing agricultural byproducts is mostly based on the biorefinery concept. The biorefinery approach involves the integration of multiple technologies to extract and utilize various valuable components from biomass, including cruciferous vegetable waste and byproducts. Technologies such as fermentation, enzymatic hydrolysis, and separation techniques are typically included in this process [41,42]. Enzymatic hydrolysis breaks down the waste biomass into fermentable sugars, which can be used for the production of biofuels, bioplastics, and bio-chemicals. Fermentation converts these sugars into high-value products such as organic acids, enzymes, and microbial proteins. Also, different separation techniques facilitate the isolation and purification of these products for further use. The developed products by biorefinery approach, such as biofuels, energy, high-value molecules, and biobased materials, including bioplastic, bio-based composites, and biopolymers, can be utilized in various non-food and food sectors [43]. 

Recently, various green extraction techniques have been explored to extract bioactive compounds from cabbage, cauliflower, and broccoli wastes, including ultrasound, microwave-assisted extraction, enzymatic methods, and isothermal pressurization cycles [44,45]. For instance, microwave-assisted extraction was optimized to maximize the total phenolic content by up to 133.57% from broccoli byproducts, showing increased efficiency compared to traditional maceration extraction methods [46]. Mostly, these methods are sustainable and cost-effective techniques for the production of cruciferous bioactive compounds.

Moreover, the synthesis of β-galactosidase was improved through the fermentation of cauliflower waste using *Enterobacter aerogenes* KCTC2190 [47]. The ideal parameters for the experiment were 40 °C, pH 7, and a cauliflower stalk concentration of 10%, following a 30 h incubation period. The maximal level of β-galactosidase production was boosted by 10% cauliflower stalk. Furthermore, the solid-state fermentation of waste cabbage was successfully conducted for extracellular enzyme production, indicating the broader potential of utilizing cruciferous vegetable wastes for fermentation processes [48]. The peak carboxymethyl cellulase (CMC) activity recorded was 817.19 IU/gds, achieved after 48 h of fermentation at 30 °C. The fermentation of broccoli waste by *Lactiplantibacillus plantarum* at 30 °C enhanced polyphenols, lactic acid, and antioxidant properties, reducing food waste and promoting probiotic benefits [49]. After the eighth day of fermentation, the fermented juice had 30.22 mg/100 mL of gallic acid, 26.89 mg/100 mL of P-coumaric acid, and 45.56 mg/100 mL of lactic acid. These studies collectively highlight the versatility and efficacy of fermenting cruciferous waste and byproducts for various valuable outputs. However, there are some drawbacks necessitating further enhancements to the process. One potential limitation associated with the fermentation of cauliflower waste is the requirement for pretreatment using a diluted phosphoric acid solution [50]. These pretreatment procedures demand additional steps in processing and equipment, potentially leading to an increase in overall production expenses. The fermentation of cabbage, cauliflower, and broccoli waste may lead to volatile fatty acid accumulation, which eventually results in problems such as foaming and low buffer capacity, which is a problem that needs to be addressed by optimizing the C/N ratio during the fermentation [51].

Anaerobic digestion provides a promising approach for valorizing biomass by converting them into valuable biogas and nutrient-rich digestate while contributing to a sustainable approach to waste utilization [52,53]. This is recognized as a biological process where the organic matter in waste and byproducts are broken down in the absence of oxygen by anaerobic microorganisms. Primarily, biogas is composed of methane and carbon dioxide. The high organic matter content of cruciferous vegetable waste could provide a rich substrate for the production of biogas which can be later used for power generation. Additionally, the residue from anaerobic digestion, called digestate, can serve as a nutrient-rich organic fertilizer for agricultural purposes. A study reported developing a large-scale novel vegetable waste recycling method involving thermostat anaerobic digestion followed by aerobic digestion, resulting in a digestate rich in N, P, and K elements; less heavy metals; and residuals of pesticides [54], while another study reported the effect of feed to microbe ratios (0.5–2.0) on the production of biogas and removal of organic matter from Chinese cabbage waste via anaerobic digestion [52]. The anaerobic co-digestion of cabbage and cauliflower waste has shown promising results, with high biodegradability, methane yield, and organic loading rates [51,55]. In contrast, high organic loading rates can reduce methane yield and energy conversion efficiency, leading to pH drops that are difficult to control during anaerobic digestion [56]. 

Pyrolysis, an advanced thermal decomposition process occurring at high temperatures (300–800 °C), produces valuable products such as biochar, bio-oil, and syngas [42,57,58]. The specific products and their proportions produced depend on various factors, including biomass type, temperature, residence time, and reactor design. Primarily, the formation mechanism of end products during pyrolysis depends on the heterogeneous composition of the biomass, making the understanding of physicochemical conversions essential to the alteration of the yield and properties of end products. As the biomass is heated to a high temperature, it undergoes thermal decomposition, and the biomass’s complex organic molecules break down into simple compounds [42]. The pyrolysis of cabbage waste demonstrated potential in the production of bioenergy and bio-chemicals, resulting in the formation of 45% condensable compounds [59]. Pyrolytic products contain high-energy chemical compounds like toluene. It has the potential to generate bioenergy with liquid pyrolytic products, accounting for more than 70% of the total energy production. The successful conversion of cabbage, cauliflower, and banana peels, as well as a combination of vegetable wastes, into biochar by the process of pyrolysis was observed, and it was found that the properties of the resulting biochar vary depending on the kind of feedstock and the temperature, indicating their potential for use as soil amendments [60]. The optimal temperature for pyrolysis was identified as approximately 400 °C, with slight variations ranging from 300 to 500 °C, depending on the specific feedstocks, and within this temperature range, biochar yields were observed to fall between 20% and 30%. Another study revealed the cabbage waste biochar produced through pyrolysis at 360 °C, having a particle size of 0.90 mm, enhances water retention in sandy soil and shows potential for improving plant growth [61]. Hence, pyrolysis emerges as a potentially advantageous approach for converting cabbage, cauliflower, and broccoli waste into useful commodities such as bioenergy, bio-chemicals, and biochar. Also, the pyrolysis of these cruciferous vegetable waste byproducts generates renewable energy in the form of biogas, which can be used for various applications. When compared to alternative approaches, using the technique of pyrolysis can be recognized as an emerging technology due to its environmentally sustainable nature, rapidity, and minimal infrastructure requirements. While the pyrolysis of cruciferous waste can produce beneficial bioproducts, it may also generate potentially hazardous pyrolysis liquids that require proper handling. Additionally, the formation of dioxins and furans like toxic byproducts is also a concern in pyrolysis processes, with their production dependent on process conditions [61].

In contrast to the advanced technologies, several conventional techniques are also employed in agricultural byproduct valorization, including composting and animal feed production. Composting can be identified as a commonly practiced, cost-effective primary method of waste valorization suitable for both small- and large-scale waste management. This process involves the microbial decomposition of waste material into organic fertilizer, which is rich in various nutrients. However, recent researchers focus on improving conventional composting systems by introducing different strategies such as co-composting, organic or inorganic additives addition, mitigation of gaseous emissions and nutrient losses, and alterations to the microbial community [62]. Another primary technique of usage of these agricultural waste and byproducts is that they can be utilized as animal feed [63]. Since these biomasses still have higher nutritional value, they are processed to increase nutritional significance and palatability or mixed with other ingredients to use as a feeding material for livestock. Moreover, some vegetable byproducts, including cruciferous byproducts, can be processed into powders, extracts, or purees, which can be further incorporated into different food formulations and other applications as natural additives which provide significant health benefits to consumers [19,64]. 

In conclusion, the valorization of cruciferous vegetable waste involves various advanced and conventional technologies, each with its specific benefits. From the biorefinery approach to anaerobic digestion and pyrolysis, these methods contribute to sustainable resource recovery and value creation, while traditional techniques like compositing and animal feed production remain viable for waste management.

**Table 3:** Different technologies involved in vegetable waste valorization.

Source	Technology Used	Treatment Conditions	Pre-Treatment	Important Facts	Research Output	Reference
Cabbage byproducts (leaves)	Biotransformation via lactic acid bacteria fermentation (*Lactiplantibacillus*, *Lacticaseibacillus*)	Mixture of 180 g cabbage leaf, 40 g rice straw, and 40 g corn flour; 5 mL of *Lactiplantibacillus plantarum* 3.3 × 10^8^ CFU/mL vacuum-packed and stored at 30 °C for 3, 7, 15, and 30 days	NA	Preserved dry matter (DM) content, decreased pH, accumulation of acetic acid, butyric acid, and ammonia positively improved microflora in final silage	Cabbage byproducts as an added value silage DM 283.4 g/kg on day 30; lactic acid 52.1 g/kg DM on day 15	[21]
Cabbage and cauliflower waste (CCW) (1:1 *w*/*w*)	Anaerobic co-digestion (mesophilic)	3 main treatment steps: start-up, inoculum acclimatisation, and treatment of the waste mixture; 65 days	CCW mixture was subjected to mechanical treatment to reduce size and stored at 4 °C until experiment	Maximum 60% biodegradability, 37 g nitrogen/kg dry weight in the residual mixture	250 mL/g VS of methane yield (VS: volatile solids)	[55]
Vegetable waste (80% broccoli, 5% cabbage, and other)	Fermentation (Fed-batch bioreactor)	Vegetable hydrolylate total sugar 300 g/L, 10 g/L yeast extract pH 6.3, dissolved oxygen 5%, 150–250 rpm	*Macerated broccoli*suspension in water (50 g/L) was treated with H_2_SO_4_ (0.5%, 1%, 3%, and 7% *v*/*v*) at 121 °C for 30 min	The most efficient strain was identified as *Enterobacter ludwigii* FMCC 204	17.6 g/L of 2,3-butanediol yield	[65]
Broccoli stalks (BS), cauliflower cores (CC)	Pyrolysis and carbonization	Heating rate of 10 °C/min; pyrolysis/carbonization temperatures of 500; 600, 700, and 800 °C, dwell time of 2 and 4 h; N_2_ and CO_2_ flows of 100 mL/min	Dried biomass particle size of 0.67 mm	Preparation of adsorbents from vegetable waste for heavy metal removal;adsorption capacity of adsorbent was effective for the removal of Cd^2+^, Zn^2+^, Ni^2+^, and Cu^2+^ from solutions; adsorption capacities were higher for adsorbents obtained from BS	Yields of obtained adsorbents from biomass pyrolysis and carbonization ranged from BS 25 to 29% and CC 26 to 30%	[66]
Vegetable waste	Thermostatic anaerobic digestion followed by thermostatic aerobic digestion	Anaerobic digestion: 37 °C, 30 days; aerobic digestion: 30 °C, 48 h	NA	Novel fast-treated recycling approach for vegetables, abundance of N, P, K, and no heavy metals in the treated waste	96% COD removal efficiency; 81.75% of Germination Index in treated biogas slurry	[54]
Cauliflower outer leaves (CL)	Fungal fermentation(filamentous fungi: *Aspergillus niger*, *A. oryzae*, *Aspergillus sojae*, *Rhizopus oryzae*, *Rhizopus azygosporus*, and *Phanerochaete chrysoporium*)	Initial inoculum 10^6^ spores/mL, mix 120 g of CL with 2 mL of inoculum in 20 mL sterilized water, fermentation at 30 °C, 7 days	NA	Fermentation facilitates releasing phenolic compounds, fermentation with *Aspergillus sojae* showed the highest level of total phenolic compounds (TPC), and kaempferol-3-O-diglucoside was dominant; fungal fermentation results in a shift in phenolic profile to a profile with less or no carbohydrate moieties at the 3-or 7-carbon position	TPC in *Aspergillus sojae* fermented samples after 1 day: 321 mg rutin equivalents (RE)/100 g fresh weight (FW)), kaempferol-3-O-diglucoside: 38–126 mg RE/100 g FW	[67]
Broccoli stems	Fermentation with *Lactiplantibacillus plantarum*	200 g of blanched samples, 2 mL of 8.6 log CFU/mL inoculum, fermentation at 37 °C for 96 h	Disruption to a maximum particle size of 5 or 10 mm followed by hot water blanching; 72 °C for 1 min	Fermentation facilitates faster drying rates and enhances phenol and flavonoid retention.	Total Phenol Content: 3.7 mg GAE/gdm. Total Flavonoid Content: 1.2 mg QE/gdm	[68]
Broccoli stalks	Solid–liquid extraction	Total fiber (TF) extraction with 80% ethanol; insoluble fiber extraction (IF) with water at a ratio of 1/2.5 (m/v), 70 °C, 30 min	Freeze drying	IF possessed a good prebiotic effect	TF and IF yields: 67% and 70%	[69]
Waste Chinese cabbage (CW)	Two-phase anaerobic digestion	Phase 1: 1 month, 0.2–0.7 kg VS·d^−1^ of CW slurry, 37 °C, 40% fresh pig manure, 20% sludge water; *Methanococcus* and *Methanosarcina* were main methanogens in anaerobic digester Phase 2: >2 months; daily feed rate of 3–11 kg CW from reactor 1	NA	In phase 2, every feed rate was used for a week, and the feed was increased steadily; higher biogas and methane productivity without nitrogen substrate and bicarbonate; fast gas production with stable operation	0.62 m^3^·(kg VS)^−1^ of biogas; 65–68% methane	[70]
Cauliflower waste	Bacteria-assisted solid–liquid extraction	Rapid solid–liquid dynamic extraction: 60 extraction cycles, with each cycle having a 3 min static phase, followed by a 2 min dynamic phase; 5 h	Enzymatic pre-treatment with *Bacillus subtilis* culture	*Bacillus subtilis* exhibits enhanced production of the essential xylanases and cellulases enzymes for breaking down plant cell walls	0.245 mg/g of polyphenol content, 0.006 g/g of isothiocyanates, 2.711 g/g of chlorogenic acid, 3.071 g/g of epigallocatechin gallate	[71]

## 4. Potential Applications of Cabbage, Broccoli, and Cauliflower Byproducts 

**Figure 2:**
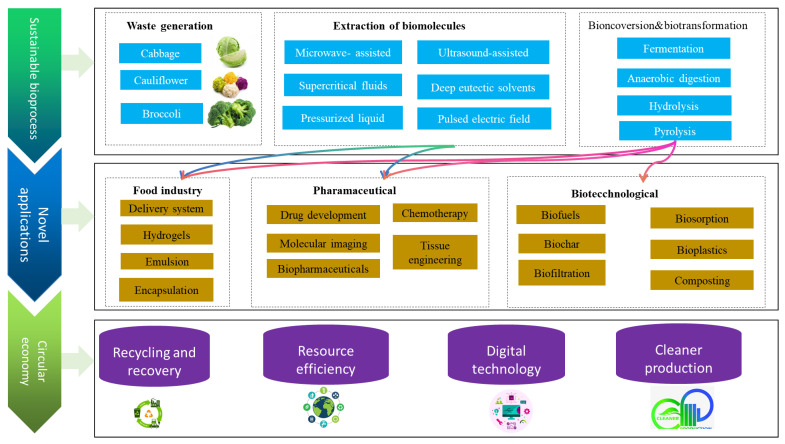
A schematic illustration of cruciferous byproducts/waste valorization.

Several potential applications can be considered for cruciferous vegetable waste/byproducts in food, pharmaceutical, and other industries. There is a growing interest in the use of these byproducts in value-added applications since many of the cruciferous vegetable byproducts, including cabbage, broccoli, and cauliflower, are still rich in beneficial nutrients and phytochemical profiles, even as a waste product. Figure 2 illustrates the overall significance of valorizing cruciferous byproducts and waste materials for different future applications while attaining circular economic goals. 

### 4.1. Food Industry Applications

#### 4.1.1. Antimicrobial Properties 

When considering foodborne diseases as well as food losses due to spoilage, there is an emerging need for effective preservation measures for foods against microbial contamination. Currently, there is an increasing demand for plant bioactive compounds with antimicrobial properties in food preservation. In this context, cruciferous byproducts demonstrated antimicrobial potential, offering an eco-friendly alternative to chemical preservatives. 

A recent study demonstrated the efficacy of pasta enriched with polyphenolic extracts from broccoli stems and leaves against *Staphylococcus* spp., *Enterobacteriaceae*, molds, and yeasts, thereby enhancing both shelf life and nutritional quality [72]. Likewise, another study confirms the potential preservative effects of broccoli seed extract through antifungal activity against the cereal contaminants, *Fusarium culmorum*, *Aspergillus niger*, and *Penicillium expansum* [73]. The compound responsible for the antifungal activity of broccoli seed extracts is an antimicrobial peptide called broccoli napin [73]. Promising antimicrobial activity of broccoli-derived compounds, such as antimicrobial peptides and sulforaphane against various foodborne pathogens, including *Staphylococcus aureus*, *Bacillus subtilis*, and *Salmonella typhimurium*, enable their application as preservatives in the functional food industry [74,75]. Moreover, research shows that glucosinolates present in cruciferous byproducts possess antifungal and antibacterial while also having bioherbicidal, antioxidant, antimutagenic, and anticarcinogenic properties [76]. Mechanisms of antimicrobial action include the inactivation of intracellular enzymes and obstruction of ATP synthesis in pathogen cells. The antimicrobial potential of cruciferous byproducts offers a promising solution to the growing concern over chemical preservatives in food. In addition to extending food shelf life and lowering the chance of foodborne infections, these byproducts can contribute to reducing the environmental impact caused by the use of chemical preservatives. By further exploring the mechanisms behind these bioactivities through further research, the immense potential of cruciferous byproducts can be employed in various applications.

#### 4.1.2. Cruciferous Component-Based Functional Delivery Systems 

The potential of cruciferous residual biomass to be used as a raw material in food-grade delivery systems has received recent research attention. Broccoli byproducts, including leaves, stalks, seeds, and roots, can be exploited as delivery vehicles for bioactive compounds or as an ingredient in food formulation. Being a rich source of dietary fiber, protein, minerals, vitamins, and phytochemicals such as sinapic acid, isochlorogenic acid, kaempferol, quercetin, and glucosinolates, makes extracts of broccoli byproducts to be incorporated as a functional ingredient in various food products such as crackers, gluten-free breads and sponge cakes, soups, and beverages enhancing their nutraceutical potentials [17,77]. Health-promoting bioactive compounds containing broccoli byproducts were used as an ingredient in developing a functional beverage [78]. To deliver the beneficial properties of glucosinolates, isothiocyanates, chlorogenic and sinapic acids, and flavonoids, broccoli byproduct extracts were enriched with a green tea matrix, and the end product was characterized by excellent phytochemical composition. Furthermore, broccoli byproducts have the potential to deliver polyunsaturated oils while preventing their degradation in the gastrointestinal tract. Recently, a group of researchers studied the potential use of broccoli byproducts as the matrix for the co-delivery of epigallocatechin gallate (EGCG) and tuna oil [32]. The delivery of bioactive compounds depends on the complexity of the carbohydrates and protein matrix in broccoli which allows the bioactive compounds to be entrapped in the matrix [17]. Therefore, there is a research opportunity to develop more sustainable delivery systems for functional foods using these cruciferous-based bioactive and functional components since they have the potential to be used in the development of food and beverage products enriched with bioactive agents, contributing to human health. However, further research is required to address concerns about safety and toxicity and to optimize the functionality and efficacy of these delivery systems.

#### 4.1.3. Other Functional Properties 

Pectin is a food additive capable of forming gels in food, pharmaceutical, and cosmetic products. Also, as a soluble dietary fiber, pectin is an important component with many beneficial health effects, including modulatory effects on gastrointestinal immune responses [79]. Commercially, they are extracted from the cell walls of different fruits and vegetables, including citrus fruits and apples. Recently, broccoli stalks were used to extract pectin as an alternative for commercial pectin production [11]. Pectin was extracted from broccoli stalks using acid extraction with an 18% yield. The obtained pectin could be effectively applied as a food-grade thickening and emulsifying agent in different food applications. 

In another study, Chinese cabbage waste was identified as a potential source of soluble dietary fiber that can be applied as a functional ingredient in beverages and nutraceutical products in the food industry [80]. Chinese cabbage waste was subjected to enzymatic hydrolysis using Celluclast 1.5 L to obtain soluble dietary fiber from the cellulose fraction of cabbages, and the obtained dietary fibers were found to have health-beneficial effects such as prebiotic, hypoglycemic, and hypolipidemic effects. Likewise, dietary fiber powder with antioxidant properties was developed from cabbage outer leaves [38]. Blanching combined with air drying at 80 °C facilitated better retention of phytochemicals in this functional powder product. 

Microwave hydrodiffusion and gravity (MHG) extracts of broccoli byproducts, including stalks, inflorescences, and leaves obtained from the frozen food industry, were evaluated for their nutritional value and bioactive compounds to use these extracts as a food ingredient [81]. MHG-assisted dehydration and extraction process preserves carbohydrates (fructose, glucose, mannitol, and pectic polysaccharides), free amino acids, glucosinolates, and other phenolic compounds in broccoli extracts. Therefore, these bioactive compound-rich extracts were incorporated in béchamel sauce as a value-added product for the food industry. 

Cauliflower waste powders were used to make flatbread to improve its nutritional (protein, crude fiber, and mineral content) and functional properties [82]. The bread could be improved by replacing wheat flour at 5–7.5% of cauliflower waste powder without influencing the sensory characteristics of the bread. The formulated bread also showed a delay in staling and microbial spoilage during storage. In another study, flour obtained from cauliflower byproducts (upper stem, stalks, and leaf midribs) was effectively used as a partial fat replacement in beef sausages [64]. Higher amounts of dietary fiber, protein, mineral, and antioxidant polyphenols in cauliflower residues improved the nutritional value of fortified food products at a low cost. Another study developed special flours from industrial byproducts of cauliflower (leaves and stalks), which can enhance the nutritional content of baked goods, especially pizza [83]. The study presented pizzas enriched with 10% newly developed flour, which showed similar characteristics to the control sample, making them suitable for both technological, industrial processing and consumer acceptance. The recovery of bioactive components in pizza depends on the portion of cauliflower byproducts used, where the stalks are the richest in glucobrassicin and the leaves have the highest carotenoid content. 

The consumer demand for natural food colorants has increased due to their potential health benefits compared to synthetic dyes. Anthocyanin is one of the natural pigments that can be extracted from fresh and agro-industrial byproducts of red cabbages, and it can be used as a color additive in different food and beverage products [84,85]. The higher thermal stability of red cabbage anthocyanin in solutions enables its intended use as a colorant in foodstuffs, while photodegradation was identified as the primary destabilizing factor [85]. The hydroethanolic anthocyanin extracts obtained from red cabbage waste could be stored under aerobic conditions in the dark at 4 °C for 20 weeks [86].

### 4.2. Medicinal Applications

Cruciferous vegetables and their derived products were identified as rich sources of bioactive compounds. The excellent chemical compositions of the underutilized parts of these vegetables have gained the attention of scientists for the implementation of cruciferous extracts in therapeutic applications. Cruciferous vegetable-based extracts can be employed in medicinal applications with potential health benefits for humans based on various pharmacological properties such as anti-inflammatory properties, anticancer activity, antimicrobial activity, antioxidant activity, hepatoprotective properties, and immunomodulatory properties [23].

#### 4.2.1. Anti-Inflammatory Properties

Cruciferous species, especially broccoli, cauliflower, and cabbage, as well as their byproducts, have proven anti-inflammatory effects with potential medicinal uses [87,88]. Bioactive substances such as isothiocyanates and glucosinolates found in cruciferous vegetables are known to have anti-inflammatory properties [89]. These substances are capable of controlling oxidant and inflammatory mediators, modifying the immune system, maintaining the integrity of the intestinal barrier, and upholding a balanced state of intestinal flora [90]. It was also indicated that these beneficial components can mitigate disorders linked to inflammation and enhance medical conditions. Moreover, preclinical and clinical evaluations of these phytochemicals proved their potential as natural anti-inflammatory drugs [89]. 

**Figure 3:**
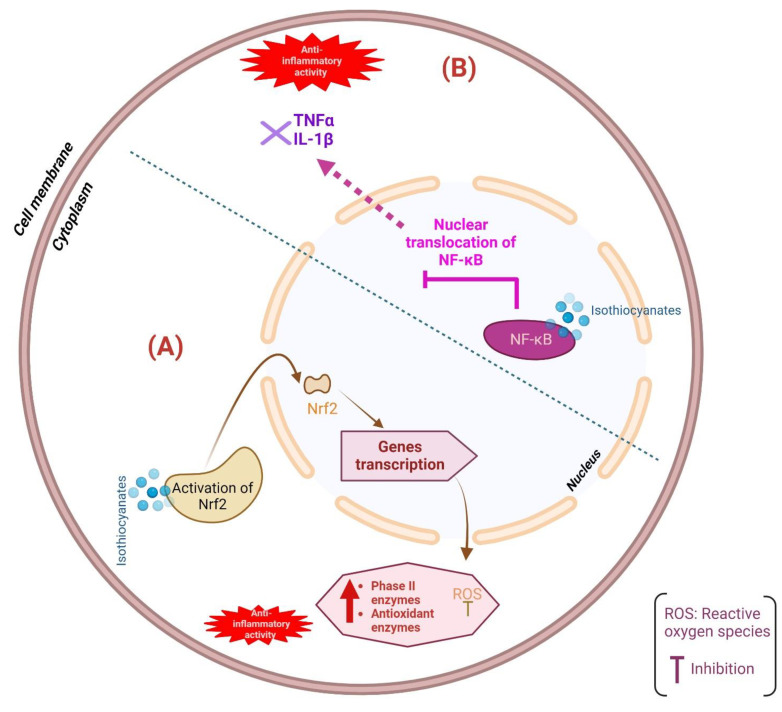
Anti-inflammatory mechanism of isothiocyanates; (**A**) modulation of Nrf2 signaling pathway, (**B**) modulation of NF-κB pathway.

Isothiocyanates are able to modulate inflammatory signaling pathways, including the nuclear factor kappa B (NF-κB) pathway and the mitogen-activated protein kinase (MAPK) pathway. Thereby, these bioactive compounds lower the production of pro-inflammatory cytokines and chemokines, which would suppress inflammation [91]. Furthermore, the bioactivity effect of isothiocyanates can be exerted by activating the redox-sensitive transcription factor nuclear factor erythroid 2-related factor 2 (Nrf2), which regulates the production of phase II and antioxidant enzymes [92]. The ability to activate Nrf2 results in isothiocyanates gaining anti-inflammatory as well as anti-carcinogenic properties. Additionally, it was demonstrated that isothiocyanates improve inflammatory phenotype in colitic mice in vivo, which plays a role in controlling inflammatory signaling pathways [89]. Figure 3 briefly illustrates the mechanism of isothiocyanates in modulating Nfr2 and NF-κB signaling pathways in showing anti-inflammatory properties. 

#### 4.2.2. Anticancer Properties 

The anticancer activity of broccoli byproducts was discovered by some researchers. Due to bioactive compounds in broccoli floret, leaf, and seed extracts, possibly glucosinolates, those broccoli extracts exhibited higher cytotoxicity effects towards different human cancer cell lines [74]. Broccoli-derived extracts, including those of the floret and sprout, were also proven to possess effective cytotoxic properties against various cancer cells in the breast, colon, ovary, and prostate due to their significantly higher sulforaphane contents [93]. Hence, these broccoli extracts can be identified as potential cancer chemopreventive agents. Also, sulforaphane in cruciferous vegetables and their byproducts is found to be an effective chemopreventive agent that possesses the potential to prevent and reverse the abnormal growth of tissues [94]. Most interestingly, it is reported that lignin extracts in their study showed lower cytotoxicity toward human bone cancer cell line, which could be applied to its anti-carcinogenic effects in therapeutic applications [95]. 

Glucosinolates, isothiocyanates, indoles, and phenolic compounds are also known to possess chemopreventive properties by effectively inhibiting cancer cells in their initiation, promotion, and progression stages. Although specific mechanisms still need to be studied in detail, generally, cancer prevention properties were demonstrated to exert, involving multiple pathways such as antioxidant activity, the modulation of detoxification enzymes, cell proliferation inhibition, cell apoptosis induction, and anti-inflammatory reactions [96]. The function of glucosinolates in cancer prevention is mainly involved in activating the Nrf2 transcription factor, inhibiting tumor necrosis factor-α (TNFα) and interleukin-1β (IL-1β), inducing apoptosis, and modulating other specific inflammatory signaling pathways in cells [97]. The anticancer efficacy of sulforaphane results from the regulation of detoxification enzymes, the inhibition of cell proliferation, apoptosis initiation, and its anti-inflammatory potential. Bioactive indoles regulate specific signaling pathways responsible for the growth and spread of cancer while the antioxidant activities as well as the anti-inflammatory properties exhibited by phenolic substances, like flavonoids and phenolic acids, also contribute to the anticancer actions. 

#### 4.2.3. Other Medicinally Beneficial Properties

Sulforaphane and glucoraphanin, which come from high-glucoraphanin broccoli, possess significant therapeutic potential. Sulforaphane is produced through metabolizing glucoraphanin, and it has anti-inflammatory, antioxidant, and immunomodulatory properties. Sulforaphane was shown to play a role in reducing inflammation, as mentioned in the above sections, as well as protecting against diseases such as diabetes, heart attacks, and cancer [54]. Incorporating glucoraphanin-enriched broccoli sprout-based supplements can enhance liver function among healthy individuals with a particular reduction in the levels of alanine aminotransferase (ALT) [98]. Additionally, melatonin can regulate the synthesis of sulforaphane and glucoraphanin and the synthesis of signal molecules such as NO and H_2_O_2_, which play a role in their synthesis mechanism [99]. Hence, the advantages of medicinal values attributed to these chemical substances include liver-boosting effects and immune response control.

According to another study, lignin was extracted from cauliflower leaf and stalk wastes and studied for their therapeutic potential [95]. Alkali (NaOH) pretreatment and Na Acetate catalyst-assisted hydro-thermal pretreatment methods under mild temperature and pressure conditions were used to extract lignin from cauliflower waste residues. The presence of aromatic and phenolic functional groups of extracted lignin is reported to be responsible for the metal-chelating and antimicrobial properties. Lignin obtained from both cauliflower leaf and stalk biomass showed effective inhibitory effects against *Bacillus subtilis* and *Staphylococcus aureus*. In another study, the industrial processing waste of broccoli was used as a promising raw material for peroxidase production, which can be implemented in different industrial and clinical applications such as enzyme immunoassays [100]. 

Industrial residues and the agricultural waste of broccoli and cauliflower waste (stems and leaves) possess bioactivities including antioxidant, anticancer, cardiovascular disease prevention, and antihypertensive properties, giving them the potential to be used in nutraceutical products [93]. 

### 4.3. Other Applications 

Despite exploring the medicinal applications, cruciferous vegetable wastes find utility in diverse areas beyond healthcare. 

Cabbage residues are found to have higher amounts of D-fructose, glucose-based fiber, and sugar. Taking advantage of that, bioethanol and a zero-calorie sweetener, which is D-psicose, were produced through hydrolysis, enzymatic conversion, and fermentation [101]. Cabbage leafy waste and cauliflower waste blended with cow dung were reported for use as the feedstock in the vermicomposting process in the presence of *Eisenia fetida* earthworm species as a sustainable and cost-effective waste conversion initiative [9]. The output vermicompost was found to have higher contents of NPK (N—49.3–85.3%, P—68.2–98.1%, K—91.8–120.3%) with lower Total Organic Carbon content (36.7–42.8%). Kimchi cabbage waste was used to produce organic acids using lactic acid bacteria (LAB) with the potential to be used as microbial nematicide [102]. The simultaneous saccharification and fermentation process was identified as the economically feasible method to produce organic acids from kimchi cabbage waste. Different organic acids, including lactic acid, fumaric acid, and acetic acid, could be efficiently produced by different LAB strains (*Lactobacillus sakei* WiKim31 and *L*. *curvatus* WiKim38), and the culture filtrate can be applied as an effective microbial nematicide at 2.5% concentration.

Recently, cauliflower and cabbage waste, including non-edible parts, were employed as heavy metal adsorbents for water pollutants [103]. Waste in the form of powder obtained through specific treatment procedures (dried at specific time–temperature combinations or chemically activated using chemical agents) from these vegetables was found to remove Pb(II) and Cd(II) heavy metals from synthetic solutions. Additionally, the pyrolysis or carbonization of cauliflower waste increased the adsorption ability to remove metal ions such as Ni^2+^, Zn^2+^, Cd^2+^, and Cu^2+^. Moreover, another research work reports potential applications of harvesting and processing waste of cabbage as a promising fermentation medium for fungal biomass production in the production of protease enzymes, bioethanol production using cabbage leafy waste, and the exploitation of cabbage waste for fuel briquette production at a reduced total cost of production [16]. 

The versatile application of cabbage, broccoli, and cauliflower byproducts extends beyond food and pharmaceuticals to include the production of biofuels, biopolymers, and the removal of heavy metals from water pollutants.

## 5. Conclusions and Future Prospects

As discussed in this review, byproducts of cruciferous vegetables, including cauliflower, broccoli, and cabbage, are known to have significant nutritional value and health benefits. Recently, there has been increasing interest in exploring the potential of utilizing these cruciferous byproducts for various industrial applications. Particularly, glucosinolates, flavonoids, anthocyanins, and carotenoids contained in these byproducts possess health-promoting effects and therapeutic effects, including antioxidant, antimicrobial, and anticancer properties, which assure their promising applicability in the pharmaceutical, cosmetic, and food industries. To advance our understanding of cruciferous byproducts, future research should delve into specific toxicological effects and assess bioavailability and metabolism. Additionally, targeted studies on potential pesticide residues and allergenicity are crucial for ensuring the safety of end products. Researchers are encouraged to explore these aspects comprehensively. Furthermore, due to the potential of cruciferous byproducts as feedstocks for biofuel production through various conversion processes, it is evident that their utilization is an opportunity to reduce dependence on fossil fuels and minimize adverse environmental impacts. 

In conclusion, the versatile application, coupled with the environmental and health benefits of cruciferous byproducts, underscore their potential to revolutionize various industries. As research continues to address safety considerations and optimize valorization processes, these byproducts are poised to play a pivotal role in sustainable and health-promoting industrial practices.

## Figures and Tables

**Table 1:** Nutritional composition of different cruciferous vegetables.

Cruciferous Vegetable	Dry Matter (DM)	Carbohydrates	Protein	Lipids	Fiber	Minerals	Reference
Broccoli (g/100 g, raw)	-	6.27	2.57	0.34	2.4	K, Ca, P: 0.303, 0.046, 0.067	[20]
Cauliflower (g/100 g, raw)	-	4.97	1.92	0.28	2	K, Ca, P: 0.299, 0.022, 0.44	
Cabbage (g/100 g, raw)	-	5.8	1.28	0.1	2.5	K, Ca, P: 0.170, 0.040, 0.020	
Cabbage leaf (g/100 g DM)	5.94 g/100 g	43.94	22	42.5	23.36	-	[21]
Chinese cabbage (g/100 g fresh weight)	-	2.20	1.50	0.20	1.00	K, Ca, Fe: 25.1, 10.5, 85.0	[5]
Kale (g/100 g fresh weight)	-	10.0	3.28	0.74	1.94	K, Ca, Fe: 44.6, 13.5, 160.0	
Arugula (g/100 g fresh weight)	-	3.65	2.58	0.66	1.60	K, Ca: 37.0, 16.0	
Brussels sprouts (g/100 g fresh weight)	-	8.67	2.55	0.51	26.94	K, Ca, Fe: 38.9, 4.2, 140.0	

## Data Availability

No new data were created or analyzed in this study. Data sharing is not applicable to this article.

## References

[1] Read Q.D., Brown S., Cuéllar A.D., Finn S.M., Gephart J.A., Marston L.T., Meyer E., Weitz K.A., Muth M.K. (2020). Assessing the environmental impacts of halving food loss and waste along the food supply chain. Sci. Total Environ..

[2] Almaraz-Sánchez I., Amaro-Reyes A., Acosta-Gallegos J.A., Mendoza-Sánchez M. (2022). Processing agroindustry by-products for obtaining value-added products and reducing environmental impact. J. Chem..

[3] Belaud J.-P., Prioux N., Vialle C., Sablayrolles C. (2019). Big data for agri-food 4.0: Application to sustainability management for by-products supply chain. Comput. Ind..

[4] Komesu A., da Silva Martins L.H., Pandey P., Kuila A., Penteado C.F.A., Penteado E.D., de Oliveira J.A.R. (2022). Fruit and Vegetable Waste an Economic Alternate to Costlier Raw Materials for Value Added Products. Waste Management.

[5] Manchali S., Chidambara Murthy K.N., Patil B.S. (2012). Crucial facts about health benefits of popular cruciferous vegetables. J. Funct. Foods.

[6] Murillo G., Mehta R.G. (2001). Cruciferous Vegetables and Cancer Prevention. Nutr. Cancer.

[7] Singh B., Sharma S., Singh B. (2010). Antioxidant enzymes in cabbage: Variability and inheritance of superoxide dismutase, peroxidase and catalase. Sci. Hortic..

[8] Zhang D., Hamauzu Y. (2004). Phenolics, ascorbic acid, carotenoids and antioxidant activity of broccoli and their changes during conventional and microwave cooking. Food Chem..

[9] Mago M., Gupta R., Yadav A., Garg V.K. (2022). Sustainable treatment and nutrient recovery from leafy waste through vermicomposting. Bioresour. Technol..

[10] Son A.-R., Kim S.-H., Valencia R.A., Jeong C.-D., Islam M., Yang C.-J., Lee S.-S. (2021). Kimchi cabbage (*Brassica rapa* L.) by-products treated with calcium oxide and alkaline hydrogen peroxide as feed ingredient for Holstein steers. J. Anim. Sci. Technol..

[11] Petkowicz C.L., Williams P.-A. (2020). Pectins from food waste: Characterization and functional properties of a pectin extracted from broccoli stalk. Food Hydrocoll..

[12] Zhang Y., Jiang Z., Wang L., Xu L. (2017). Extraction optimization, antioxidant, and hypoglycemic activities in vitro of polysaccharides from broccoli byproducts. J. Food Biochem..

[13] Thomas M., Badr A., Desjardins Y., Gosselin A., Angers P. (2018). Characterization of industrial broccoli discards (*Brassica oleracea* var. italica) for their glucosinolate, polyphenol and flavonoid contents using UPLC MS/MS and spectrophotometric methods. Food Chem..

[14] Xu Y., Li Y., Bao T., Zheng X., Chen W., Wang J. (2017). A recyclable protein resource derived from cauliflower by-products: Potential biological activities of protein hydrolysates. Food Chem..

[15] Chaisamlitpol S., Hiranvarachat B., Srichumpoung J., Devahastin S., Chiewchan N. (2014). Bioactive compositions of extracts from cabbage outer leaves as affected by drying pretreatment prior to microwave-assisted extraction. Sep. Purif. Technol..

[16] Moreb N., Murphy A., Jaiswal S., Jaiswal A.K. (2020). Cabbage. Nutritional Composition and Antioxidant Properties of Fruits and Vegetables.

[17] Li H., Xia Y., Liu H.-Y., Guo H., He X.-Q., Liu Y., Wu D.-T., Mai Y.-H., Li H.-B., Zou L. (2022). Nutritional values, beneficial effects, and food applications of broccoli (*Brassica oleracea* var. italica Plenck). Trends Food Sci. Technol..

[18] Schäfer J., Stanojlovic L., Trierweiler B., Bunzel M. (2017). Storage related changes of cell wall based dietary fiber components of broccoli (*Brassica oleracea* var. italica) stems. Food Res. Int..

[19] Tlais A.Z.A., Fiorino G.M., Polo A., Filannino P., Di Cagno R. (2020). High-Value Compounds in Fruit, Vegetable and Cereal Byproducts: An Overview of Potential Sustainable Reuse and Exploitation. Molecules.

[20] Ekenci D., Yılmaz B., Capasso R., Ozer D. (2022). Cruciferous Vegetables and Their Bioactive Metabolites: From Prevention to Novel Therapies of Colorectal Cancer. Evid.-Based Complement. Altern. Med..

[21] Du G., Zhang G., Shi J., Zhang J., Ma Z., Liu X., Yuan C., Li X., Zhang B. (2021). Keystone taxa *Lactiplantibacillus* and *Lacticaseibacillus* directly improve the ensiling performance and microflora profile in co-ensiling cabbage byproduct and rice straw. Microorganisms.

[22] Kovalikova Z., Kubes J., Skalicky M., Kuchtickova N., Maskova L., Tuma J., Vachova P., Hejnak V. (2019). Changes in content of polyphenols and ascorbic acid in leaves of white cabbage after pest infestation. Molecules.

[23] Favela-González K.M., Hernández-Almanza A.Y., De la Fuente-Salcido N.M. (2020). The value of bioactive compounds of cruciferous vegetables (*Brassica*) as antimicrobials and antioxidants: A review. J. Food Biochem..

[24] Choi S.-H., Park S., Lim Y.P., Kim S.-J., Park J.-T., An G. (2014). Metabolite Profiles of Glucosinolates in Cabbage Varieties (*Brassica oleracea* var. capitata) by Season, Color, and Tissue Position Introduction. Environ. Biotechnol..

[25] Llorach R., Gil-Izquierdo A., Ferreres F., Tomás-Barberán F.A. (2003). HPLC-DAD-MS/MS ESI characterization of unusual highly glycosylated acylated flavonoids from cauliflower (*Brassica oleracea* L. var. botrytis) agroindustrial byproducts. J. Agric. Food Chem..

[26] Wijngaard H.H., Rößle C., Brunton N. (2009). A survey of Irish fruit and vegetable waste and by-products as a source of polyphenolic antioxidants. Food Chem..

[27] Zanoni F., Primiterra M., Angeli N., Zoccatelli G. (2020). Microencapsulation by spray-drying of polyphenols extracted from red chicory and red cabbage: Effects on stability and color properties. Food Chem..

[28] Amofa-Diatuo T., Anang D.M., Barba F.J., Tiwari B.K. (2017). Development of new apple beverages rich in isothiocyanates by using extracts obtained from ultrasound-treated cauliflower by-products: Evaluation of physical properties and consumer acceptance. J. Food Compos. Anal..

[29] Xu Y., Bao T., Han W., Chen W., Zheng X., Wang J. (2016). Purification and identification of an angiotensin I-converting enzyme inhibitory peptide from cauliflower by-products protein hydrolysate. Process Biochem..

[30] Gonzales G.B., Raes K., Coelus S., Struijs K., Smagghe G., Van Camp J. (2014). Ultra(high)-pressure liquid chromatography-electrospray ionization-time-of-flight-ion mobility-high definition mass spectrometry for the rapid identification and structural characterization of flavonoid glycosides from cauliflower waste. J. Chromatogr. A.

[31] Salas-Millán J.-Á., Aznar A., Conesa E., Conesa-Bueno A., Aguayo E. (2022). Functional food obtained from fermentation of broccoli by-products (stalk): Metagenomics profile and glucosinolate and phenolic compounds characterization by LC-ESI-QqQ-MS/MS. LWT.

[32] Shi M., Ying D.Y., Ye J.H., Sanguansri L., Augustin M.A. (2020). Broccoli byproducts for protection and co-delivery of EGCG and tuna oil. Food Chem..

[33] Cruz A.B., Pitz H.d.S., Veber B., Bini L.A., Maraschin M., Zeni A.L.B. (2016). Assessment of bioactive metabolites and hypolipidemic effect of polyphenolic-rich red cabbage extract. Pharm. Biol..

[34] Gudiño I., Martín A., Casquete R., Prieto M.H., Ayuso M.C., Córdoba M.d.G. (2022). Evaluation of Broccoli (*Brassica oleracea* VAR. Italica) Crop By-Products as Sources of Bioactive Compounds. SSRN Electron. J..

[35] Formica-Oliveira A.C., Martínez-Hernández G.B., Díaz-López V., Artés F., Artés-Hernández F. (2017). Use of postharvest UV-B and UV-C radiation treatments to revalorize broccoli byproducts and edible florets. Innov. Food Sci. Emerg. Technol..

[36] Kowalski A., Agati G., Grzegorzewska M., Kosson R., Kusznierewicz B., Chmiel T., Bartoszek A., Tuccio L., Grifoni D., Vågen I.M. (2021). Valorization of waste cabbage leaves by postharvest photochemical treatments monitored with a non-destructive fluorescence-based sensor. J. Photochem. Photobiol. B Biol..

[37] Martínez-Zamora L., Castillejo N., Artés-Hernández F. (2021). UV-B radiation as abiotic elicitor to enhance phytochemicals and development of red cabbage sprouts. Horticulturae.

[38] Tanongkankit Y., Chiewchan N., Devahastin S. (2010). Effect of processing on antioxidants and their activity in dietary fiber powder from cabbage outer leaves. Dry. Technol..

[39] Katsube T., Tsurunaga Y., Sugiyama M., Furuno T., Yamasaki Y. (2009). Effect of air-drying temperature on antioxidant capacity and stability of polyphenolic compounds in mulberry (*Morus alba* L.) leaves. Food Chem..

[40] Mrkìc V., Cocci E., Rosa M.D., Sacchetti G. (2006). Effect of drying conditions on bioactive compounds and antioxidant activity of broccoli (*Brassica oleracea* L.). J. Sci. Food Agric..

[41] Abdul P.M., Jahim J.M., Harun S., Markom M., Lutpi N.A., Hassan O., Balan V., Dale B.E., Mohd Nor M.T. (2016). Effects of changes in chemical and structural characteristic of ammonia fibre expansion (AFEX) pretreated oil palm empty fruit bunch fibre on enzymatic saccharification and fermentability for biohydrogen. Bioresour. Technol..

[42] Kumar V., Sharma N., Umesh M., Selvaraj M., Al-Shehri B.M., Chakraborty P., Duhan L., Sharma S., Pasrija R., Awasthi M.K. (2022). Emerging challenges for the agro-industrial food waste utilization: A review on food waste biorefinery. Bioresour. Technol..

[43] Sharma P., Gaur V.K., Kim S.-H., Pandey A. (2020). Microbial strategies for bio-transforming food waste into resources. Bioresour. Technol..

[44] Artés-Hernández F., Martínez-Zamora L., Cano-Lamadrid M., Hashemi S., Castillejo N. (2023). Genus Brassica By-Products Revalorization with Green Technologies to Fortify Innovative Foods: A Scoping Review. Foods.

[45] González F., Quintero J., Del Río R., Mahn A. (2021). Optimization of an extraction process to obtain a food-grade sulforaphane-rich extract from broccoli (*Brassica oleracea* var. italica). Molecules.

[46] Rodríguez García S.L., Raghavan V. (2022). Microwave-assisted extraction of phenolic compounds from broccoli (*Brassica oleracea*) stems, leaves, and florets: Optimization, characterization, and comparison with maceration extraction. Recent Prog. Nutr..

[47] Maity M., Bhattacharyya D.K., Bhowal J. (2023). Improvement of β-galactosidase production by solid-state fermentation using cauliflower (*Brassica oleraceae* var. botrytis) waste by Enterobacter aerogenes KCTC2190. Res. J. Biotechnol..

[48] Das A., Ghosh U. (2009). Solid-state fermentation of waste cabbage by *Penicillium notatum* NCIM NO-923 for production and characterization of cellulases. J. Sci. Ind. Res..

[49] Zdziobek P., Jodłowski G.S., Strzelec E.A. (2023). Biopreservation and Bioactivation Juice from Waste Broccoli with *Lactiplantibacillus plantarum*. Molecules.

[50] Majumdar S., Naha A., Bhattacharyya D., Bhowal J. (2019). Effective delignification and decrystallization of cauliflower wastes by using dilute phosphoric acid for efficient enzymatic digestibility to produce fermentable sugars. Biomass Bioenergy.

[51] Beniche I., Hungría J., El Bari H., Siles J., Chica A., Martín M. (2021). Effects of C/N ratio on anaerobic co-digestion of cabbage, cauliflower, and restaurant food waste. Biomass Convers. Biorefinery.

[52] Kafle G.K., Bhattarai S., Kim S.H., Chen L. (2014). Effect of feed to microbe ratios on anaerobic digestion of Chinese cabbage waste under mesophilic and thermophilic conditions: Biogas potential and kinetic study. J. Environ. Manag..

[53] Pramanik S.K. (2022). Anaerobic co-digestion of municipal organic solid waste: Achievements and perspective. Bioresour. Technol. Rep..

[54] Li J., Wan D., Jin S., Ren H., Wang Y., Huang J., Li H., Zhang G. (2023). Fast treatment and recycling method of large-scale vegetable wastes. Sci. Total Environ..

[55] Beniche I., El Bari H., Siles J.A., Chica A.F., Martín M.Á. (2021). Methane production by anaerobic co-digestion of mixed agricultural waste: Cabbage and cauliflower. Environ. Technol..

[56] Azevedo A., Lapa N., Moldão M., Duarte E. (2023). Opportunities and challenges in the anaerobic co-digestion of municipal sewage sludge and fruit and vegetable wastes: A review. Energy Nexus.

[57] Kim S., Lee Y., Andrew Lin K.Y., Hong E., Kwon E.E., Lee J. (2020). The valorization of food waste via pyrolysis. J. Clean. Prod..

[58] Zhao J., Wang Z., Li J., Yan B., Chen G. (2022). Pyrolysis of food waste and food waste solid digestate: A comparative investigation. Bioresour. Technol..

[59] Tahir M.H., Mubashir T., Schulze M., Irfan R.M. (2022). Thermochemical conversion of cabbage waste to bioenergy and bio-chemicals production. Int. J. Energy Res..

[60] Pradhan S., Abdelaal A.H., Mroue K., Al-Ansari T., Mackey H.R., McKay G. (2020). Biochar from vegetable wastes: Agro-environmental characterization. Biochar.

[61] Pradhan S., Shahbaz M., Abdelaal A., Al-Ansari T., Mackey H.R., McKay G. (2020). Optimization of process and properties of biochar from cabbage waste by response surface methodology. Biomass Convers. Biorefinery.

[62] Awasthi S.K., Sarsaiya S., Awasthi M.K., Liu T., Zhao J., Kumar S., Zhang Z. (2020). Changes in global trends in food waste composting: Research challenges and opportunities. Bioresour. Technol..

[63] Mahmoud Y. (2016). Using Broccoli Plant Wastes in Sheep Rations. Egypt. J. Nutr. Feed..

[64] Abul-Fadl M.M. (2012). Nutritional and chemical evaluation of white cauliflower by-products flour and the effect of its addition on beef sausage quality. J. Appl. Sci. Res..

[65] Liakou V., Pateraki C., Palaiogeorgou A.M., Kopsahelis N., Machado de Castro A., Guimarães Freire D.M., Nychas G.J.E., Papanikolaou S., Koutinas A. (2018). Valorisation of fruit and vegetable waste from open markets for the production of 2,3-butanediol. Food Bioprod. Process..

[66] Landin-Sandoval V.J., Mendoza-Castillo D.I., Bonilla-Petriciolet A., Aguayo-Villarreal I.A., Reynel-Avila H.E., Gonzalez-Ponce H.A. (2020). Valorization of agri-food industry wastes to prepare adsorbents for heavy metal removal from water. J. Environ. Chem. Eng..

[67] Huynh N.T., Smagghe G., Gonzales G.B., Van Camp J., Raes K. (2016). Extraction and bioconversion of kaempferol metabolites from cauliflower outer leaves through fungal fermentation. Biochem. Eng. J..

[68] Bas-Bellver C., Barrera C., Betoret N., Seguí L. (2023). Impact of Fermentation Pretreatment on Drying Behaviour and Antioxidant Attributes of Broccoli Waste Powdered Ingredients. Foods.

[69] Núñez-Gómez V., González-Barrio R., Baenas N., Moreno D.A., Periago M.J. (2022). Dietary-fibre-rich fractions isolated from broccoli stalks as a potential functional ingredient with phenolic compounds and glucosinolates. Int. J. Mol. Sci..

[70] Dong X., Shao L., Wang Y., Kou W., Cao Y., Zhang D. (2015). Biogas by two-stage microbial anaerobic and semi-continuous digestion of Chinese cabbage waste. Chin. J. Chem. Eng..

[71] Doria E., Buonocore D., Marra A., Bontà V., Gazzola A., Dossena M., Verri M., Calvio C. (2022). Bacterial-Assisted Extraction of Bioactive Compounds from Cauliflower. Plants.

[72] Angiolillo L., Spinelli S., Conte A., Del Nobile M.A. (2019). Extract from broccoli byproducts to increase fresh filled pasta shelf life. Foods.

[73] Thery T., Lynch K.M., Zannini E., Arendt E.K. (2020). Isolation, characterisation and application of a new antifungal protein from broccoli seeds—New food preservative with great potential. Food Control.

[74] Le T.N., Sakulsataporn N., Chiu C.-H., Hsieh P.-C. (2020). Polyphenolic profile and varied bioactivities of processed Taiwanese grown broccoli: A comparative study of edible and non-edible parts. Pharmaceuticals.

[75] Pacheco-Cano R.D., Salcedo-Hernández R., Casados-Vázquez L.E., Wrobel K., Bideshi D.K., Barboza-Corona J.E. (2020). Class I defensins (BraDef) from broccoli (*Brassica oleracea* var. italica) seeds and their antimicrobial activity. World J. Microbiol. Biotechnol..

[76] Vig A.P., Rampal G., Thind T.S., Arora S. (2009). Bio-protective effects of glucosinolates—A review. LWT-Food Sci. Technol..

[77] Alvarez-Jubete L., Valverde J., Kehoe K., Reilly K., Rai D.K., Barry-Ryan C. (2014). Development of a Novel Functional Soup Rich in Bioactive Sulforaphane Using Broccoli (*Brassica oleracea* L. ssp. italica) Florets and Byproducts. Food Bioprocess Technol..

[78] Dominguez-Perles R., Moreno D.A., Carvajal M., Garcia-Viguera C. (2011). Composition and antioxidant capacity of a novel beverage produced with green tea and minimally-processed byproducts of broccoli. Innov. Food Sci. Emerg. Technol..

[79] Beukema M., Faas M.M., de Vos P. (2020). The effects of different dietary fiber pectin structures on the gastrointestinal immune barrier: Impact via gut microbiota and direct effects on immune cells. Exp. Mol. Med..

[80] Park S.Y., Yoon K.Y. (2015). Enzymatic production of soluble dietary fiber from the cellulose fraction of Chinese cabbage waste and potential use as a functional food source. Food Sci. Biotechnol..

[81] Ferreira S.S., Passos C.P., Cardoso S.M., Wessel D.F., Coimbra M.A. (2018). Microwave assisted dehydration of broccoli by-products and simultaneous extraction of bioactive compounds. Food Chem..

[82] saleh s. (2022). Quality Attributes of Shamy Bread Supplemented with Cauliflower Wastes. Egypt. J. Food Sci..

[83] Nartea A., Fanesi B., Pacetti D., Lenti L., Fiorini D., Lucci P., Frega N.G., Falcone P.M. (2023). Cauliflower by-products as functional ingredient in bakery foods: Fortification of pizza with glucosinolates, carotenoids and phytosterols. Curr. Res. Food Sci..

[84] Ayala-Zavala J., Vega-Vega V., Rosas-Domínguez C., Palafox-Carlos H., Villa-Rodriguez J., Siddiqui M.W., Dávila-Aviña J., González-Aguilar G. (2011). Agro-industrial potential of exotic fruit byproducts as a source of food additives. Food Res. Int..

[85] Dyrby M., Westergaard N., Stapelfeldt H. (2001). Light and heat sensitivity of red cabbage extract in soft drink model systems. Food Chem..

[86] Patras A. (2019). Stability and colour evaluation of red cabbage waste hydroethanolic extract in presence of different food additives or ingredients. Food Chem..

[87] Cicio A., Serio R., Zizzo M.G. (2022). Anti-inflammatory potential of Brassicaceae-derived phytochemicals: In vitro and in vivo evidence for a putative role in the prevention and treatment of IBD. Nutrients.

[88] Nisar A., Jagtap S., Deshpande M., Harsulkar A., Ranjekar P., Prakash O. (2023). Phytochemicals in the treatment of inflammation-associated diseases: The journey from preclinical trials to clinical practice. Front. Pharmacol..

[89] Sturm C., Wagner A.E. (2017). Brassica-derived plant bioactives as modulators of chemopreventive and inflammatory signaling pathways. Int. J. Mol. Sci..

[90] Deng W., Du H., Liu D., Ma Z. (2022). The role of natural products in chronic inflammation. Front. Pharmacol..

[91] Park H.-J., Kim S.-J., Park S.-J., Eom S.-H., Gu G.-J., Kim S.H., Youn H.-S. (2013). Phenethyl isothiocyanate regulates inflammation through suppression of the TRIF-dependent signaling pathway of Toll-like receptors. Life Sci..

[92] Ernst I.M., Wagner A.E., Schuemann C., Storm N., Höppner W., Döring F., Stocker A., Rimbach G. (2011). Allyl-, butyl- and phenylethyl-isothiocyanate activate Nrf2 in cultured fibroblasts. Pharmacol. Res..

[93] Paśko P., Tyszka-Czochara M., Galanty A., Gdula-Argasińska J., Żmudzki P., Bartoń H., Zagrodzki P., Gorinstein S. (2018). Comparative study of predominant phytochemical compounds and proapoptotic potential of broccoli sprouts and florets. Plant Foods Hum. Nutr..

[94] Kaiser A.E., Baniasadi M., Giansiracusa D., Giansiracusa M., Garcia M., Fryda Z., Wong T.L., Bishayee A. (2021). Sulforaphane: A broccoli bioactive phytocompound with cancer preventive potential. Cancers.

[95] Majumdar S., Seth S., Bhattacharyya D.K., Bhowal J. (2021). Evaluation of Therapeutic Properties of Lignins Extracted from Cauliflower Wastes for Their Potent Valorization Through Sustainable Approach. Waste Biomass Valorization.

[96] Mandrich L., Caputo E. (2020). Brassicaceae-derived anticancer agents: Towards a green approach to beat cancer. Nutrients.

[97] Orouji N., Asl S.K., Taghipour Z., Habtemariam S., Nabavi S.M., Rahimi R. (2023). Glucosinolates in cancer prevention and treatment: Experimental and clinical evidence. Med. Oncol..

[98] Satomi S., Takahashi S., Yoshida K., Shimizu S., Inoue T., Takara T., Suganuma H. (2022). Effects of broccoli sprout supplements enriched in glucoraphanin on liver functions in healthy middle-aged adults with high-normal serum hepatic biomarkers: A randomized controlled trial. Front. Nutr..

[99] Ma S., Bao J., Lu Y., Lu X., Tian P., Zhang X., Yang J., Shi X., Pu Z., Li S. (2022). Glucoraphanin and sulforaphane biosynthesis by melatonin mediating nitric oxide in hairy roots of broccoli (*Brassica oleracea* L. var. italica Planch): Insights from transcriptome data. BMC Plant Biol..

[100] Duarte-Vázquez M.A., García-Padilla S., García-Almendárez B.E., Whitaker J.R., Regalado C. (2007). Broccoli processing wastes as a source of peroxidase. J. Agric. Food Chem..

[101] Song Y., Nguyen Q.A., Wi S.G., Yang J., Bae H.J. (2017). Strategy for dual production of bioethanol and D-psicose as value-added products from cruciferous vegetable residue. Bioresour. Technol..

[102] Kim H.M., Park J.H., Choi I.S., Wi S.G., Ha S., Chun H.H., Hwang I.M., Chang J.Y., Choi H.-J., Kim J.-C. (2018). Effective approach to organic acid production from agricultural kimchi cabbage waste and its potential application. PLoS ONE.

[103] Matei E., Râpă M., Predescu A.M., Țurcanu A.A., Vidu R., Predescu C., Bobirica C., Bobirica L., Orbeci C. (2021). Valorization of Agri-Food Wastes as Sustainable Eco-Materials for Wastewater Treatment: Current State and New Perspectives. Materials.

